# 1980. A Case Series of Serratia Marcescens Endocarditis in Persons Who Inject Drugs

**DOI:** 10.1093/ofid/ofac492.1605

**Published:** 2022-12-15

**Authors:** Julia Christine Cook, Matthew B Anderson, Erin W Barnes

**Affiliations:** Atrium Health Wake Forest Baptist Medical Center, Winston Salem, North Carolina; Atrium Health Wake Forest Baptist, Winston Salem, North Carolina; Atrium Health Wake Forest Baptist, Winston Salem, North Carolina

## Abstract

**Background:**

Serratia marcescens is a gram-negative bacillus that is widespread in the environment and has been recognized as a rare cause of infective endocarditis (IE), particularly in persons who inject drugs (PWID). The literature consists of a 19 person case series in the 1970s followed by just a handful of case reports and small case series. At AHWFB (Atrium Health Wake Forest Baptist), we have noticed an increasing number of severe infections, including IE, with Serratia marcescens in PWID.

**Methods:**

We conducted a retrospective chart review of all cases of Serratia grown from sterile culture sites in the AHWFB system between 8/1/2016 -10/31/2021. Charts were reviewed to confirm status of injection drug use in the 3 months prior to admission, ≥18 years old, and possible or definite IE by Modified Duke’s criteria.

**Results:**

Twenty two participants were included in the study. The median age was 29.5 years. Eighteen (90%) were white and 16 (73%) were female. Sixteen (73%) had injection of opioids and 3 (14%) had injection of stimulants clearly indicated. Ten (45.5%) had stimulant use documented but the route was not clear. Nineteen (95%) had evidence of active or resolved HCV infection. The median duration of bacteremia was 2 days. Two cases of IE involved the aortic valve, 8 mitral, 3 pulmonic, and 10 tricuspid. Four (18%) of the participants had prosthetic valve IE. Nine (41%) of the participants had a history of previous IE episode. Eighteen participants had evidence of septic emboli: 10 pulmonary, 7 cerebral, and 5 major arterial. In terms of disposition, 4 (18%) patients died, 4 left against medical advice, 12 (54%) remained at a facility for the duration of their antibiotics. One patient was transferred to another facility and one was discharged to complete antibiotic therapy via peripheral IVs in an infusion center. Two patients expired before a final antibiotic regimen could be determined. Ten participants were treated with beta lactam monotherapy, 1 with fluoroquinolone monotherapy, and 9 with dual therapy with a beta lactam and fluoroquinolone.

**Conclusion:**

Serratia marcescens, a rare cause of IE, has been found in a population of PWID in a North Carolina hospital system. More research is necessary to understand why this unusual infection is clustering in this population and the optimal treatment regimen.

**Disclosures:**

**All Authors**: No reported disclosures.

